# Our Position on the Gold Question

**Published:** 1855-07

**Authors:** 


### OUR POSITION ON THE GOLD QUESTION.

The subject of crystal gold receives at present, a large share of attention from the profession. A laudable desire for knowledge on the part of some, yankee curiosity with others, and the ———of a third class, give rise to a vast amount of inquisitiveness on the subject. Privately and publicly, by friend and by foe, we are reminded of our "promise," to publish our views on the crystalization of gold.

Well, in the language of the nursery, we say "don't teaze." Our promise had reference only to certain circumstances and conditions, and *no reference at all to time.* Without abandoning our position then, we cannot publish till the conditions are fulfilled and the circumstances take place. It may be true that many members of the profession expected an earlier fulfilment of the conditions and are consequently disappointed. Well, we too are disappointed in the results of some of our experiments, and in not being able, as yet, to try others, and if they feel any worse about it than we do, we'll adopt their feelings and forego our own.

But there is another view of the case. We have been frequently advised, by professional brethren whose opinions we respect, to withhold our experence till the question of the practical utility of crystal gold is settled. Their argument is this: By the publication, we would induce many, if not all who fancy themselves chemists, to attempt the manufacture of the article, and thus the profession would be flooded with spurious preparations, and the question would be decided, not on its real merits, but on the qualities of the majority of the specimens in market. If this view of the case be correct, (and it is a plausible one,) a promise to publish *now*, even if we had made it, would be better broken, than kept.

When we began to experiment for crystal gold, we soon obtained an article that we preferred to foil for many cavities. we experimented carefully with it, and a few of our friends did the same. The result was a small demand for the article, and we sent it out, aiming always to introduce it only to the better class of operators. This we conceived to be the best, and the only method, of testing its utility. We endeavored to say and write just enough to call attention to it, and have made no great effort to introduce it. On the contrary, we have declined to furnish it, in many cases, when ordered.

In conclusion, we intend patiently to continue our experiments as time and opportunity will permit. We are neither sanguine nor excited on the subject. We have not, as yet, derived a pecuniary profit from our manufacture of crystal gold, nor do we expect to. We can make better fillings with it than *we* can with foil. If the same should prove true with a large portion of the profession, it will then be time to make arrangements for its extensive manufacture. Such arrangements we could not personally carry out, without abandoning our profession, and we harbor no such thoughts at present. In the mean time, if a better article than ours is produced, we will use it, if obtainable, and abandon our own.

TAFT & WATT.

